# Prevalence of Multimorbidity and Chronic Diseases in citizens of the Métis Nation of Ontario

**DOI:** 10.23889/ijpds.v9i5.2543

**Published:** 2024-09-18

**Authors:** Noel Tsui, Shelley Cripps, Abigail Simms, Gabriel Tjong, Haley Golding, Sarah Edwards

**Affiliations:** 1 ICES Central; 2 Métis Nation of Ontario

## Objective and Approach

Population-based analyses of Métis health in Canada are limited. The Métis are a distinct Indigenous people with both First Nation and Euro-Settler ancestry. This study aimed to examine multimorbidity in the Métis Nation of Ontario (MNO) citizens from 2009 to 2019. Registered MNO citizens were linked to Ontario’s administrative health data. An existing data algorithm identified individuals with multimorbidity (2+ chronic conditions) among MNO citizens and the Ontario population aged 18+. Annual prevalence rates of multimorbidity were calculated and rates in the latest year were compared across income quintiles, age, and sex.

## Results & Conclusion

The prevalence of multimorbidity increased in MNO citizens from 49.2 per 100 (CI: 48.1-50.2) in 2009 to 61.0 per 100 (CI: 59.9-62.1) in 2018. A consistently higher prevalence rate was seen in MNO citizens compared to non-MNO citizens across the entire period. Female MNO citizens had a higher prevalence of multimorbidity compared to males. The prevalence of multimorbidity increased with age in MNO citizens from 40.5 per 100 (CI: 39.1-41.8) in those aged 18 to 44 years up to 91.6 per 100 (CI: 88.4-98.0) in those aged 65 years and older. An income gradient was evident with MNO citizens in the highest income quintile having the lowest prevalence of multimorbidity (57.1 per 100) compared to those in the lowest income quintile (66.3 per 100).

## Implications

Understanding the burden of multimorbidity in MNO citizens is essential for the MNO to guide program and policy planning, as well as support decision-making related to resource allocation.

